# Remarkable Vitality in a New-born Child

**Published:** 1868-11

**Authors:** 


Remarkable Vitality in a New-born Child.—Dr. James T. Newman records in the *Chicago Medical Journal*, Oct. 1, 1868, the following extraordinary instance of vitality in a new-born child:—

"At half-past twelve in the evening he saw, on account of hemorrhage, a young woman who had recently given birth to a child. The friends were trying to conceal her shame. It had been 'born at 8 o'clock in the morning, and was quietly wrapped up in an old blanket and put out of sight.' He was told that it was still-born. At his request the child was shown him, and there was something in its face told him it was not dead, but he said nothing. When he made his visit in the morning, the child was in the coffin. Upon request, it was again shown to him, and to his astonishment, upon applying a stethoscope, he could distinctly hear the sound of the heart. He took the child out of the coffin, used Marshall Hall's method, and in the course of thirty minutes the child commenced breathing; the pulse was natural; it cried, and took the breast eagerly. It is a fine-looking boy to-day, and for aught I know, bids fair to live three score and ten years."

The doctor does not tell us why he did not attempt to resussitate the child when he first saw it.—*Humboldt Medical Archives.*—*Boston Med. Journal.*

